# Atrial SERCA2a Overexpression Has No Affect on Cardiac Alternans but Promotes Arrhythmogenic SR Ca^2+^ Triggers

**DOI:** 10.1371/journal.pone.0137359

**Published:** 2015-09-09

**Authors:** Michelle M. J. Nassal, Xiaoping Wan, Kenneth R. Laurita, Michael J. Cutler

**Affiliations:** 1 Heart and Vascular Research Center, Case Western Reserve University, Cleveland, OH, United States of America; 2 Department of Physiology and Biophysics, Case Western Reserve University, Cleveland, OH, United States of America; 3 Intermountain Heart Institute, Salt Lake City, Utah, United States of America; Baylor College of Medicine, UNITED STATES

## Abstract

**Background:**

Atrial fibrillation (AF) is the most common arrhythmia in humans, yet; treatment has remained sub-optimal due to poor understanding of the underlying mechanisms. Cardiac alternans precede AF episodes, suggesting an important arrhythmia substrate. Recently, we demonstrated ventricular SERCA2a overexpression suppresses cardiac alternans and arrhythmias. Therefore, we hypothesized that atrial SERCA2a overexpression will decrease cardiac alternans and arrhythmias.

**Methods:**

Adult rat isolated atrial myocytes where divided into three treatment groups 1) Control, 2) SERCA2a overexpression (Ad.SERCA2a) and 3) SERCA2a inhibition (Thapsigargin, 1μm). Intracellular Ca^2+^ was measured using Indo-1AM and Ca^2+^ alternans (Ca-ALT) was induced with a standard ramp pacing protocol.

**Results:**

As predicted, SR Ca^2+^ reuptake was enhanced with SERCA2a overexpression (p< 0.05) and reduced with SERCA2a inhibition (p<0.05). Surprisingly, there was no difference in susceptibility to Ca-ALT with either SERCA2a overexpression or inhibition when compared to controls (p = 0.73). In contrast, SERCA2a overexpression resulted in increased premature SR Ca2+ (SCR) release compared to control myocytes (28% and 0%, p < 0.05) and concomitant increase in SR Ca^2+^ load (p<0.05). Based on these observations we tested *in-vivo* atrial arrhythmia inducibility in control and Ad.SERCA2a animals using an esophageal atrial burst pacing protocol. There were no inducible atrial arrhythmias in Ad.GFP (n = 4) animals though 20% of Ad.SERCA2a (n = 5) animals had inducible atrial arrhythmias (p = 0.20).

**Conclusions:**

Our findings suggest that unlike the ventricle, SERCA2a is not a key regulator of cardiac alternans in the atrium. Importantly, SERCA2a overexpression in atrial myocytes can increase SCR, which may be arrhythmogenic.

## Introduction

Atrial fibrillation (AF) is the most common arrhythmia in humans,[1] yet current treatment options for AF have suboptimal efficacy. The limitations of current treatments for atrial fibrillation are likely secondary to incomplete understanding of the mechanisms underlying the development of atrial fibrillation. This emphasizes the need for better understanding the mechanisms of AF in order to develop more mechanism-directed AF therapies.

Myocardial calcium cycling dysregulation is an important mechanism for arrhythmogenic cardiac alternans in the ventricle and is a postulated mechanism of AF.[2,3] Mathematical models have predicted cardiac alternans as an important mechanism for the development of AF and perhaps more important, recent data in humans has shown that cardiac alternans precedes the onset of AF.[4–7]

We previously established that SERCA2a expression is an important regulator of calcium-alternans (Ca-ALT) in the ventricle.[8] Specifically, we found that increased SERCA2a expression in both the normal and failing ventricle increases the HR threshold (i.e. decreased susceptibility) for Ca-ALT with a concomitant reduction in inducible ventricular arrhythmias. Based on these data we hypothesized that SERCA2a overexpression will decrease cardiac alternans and arrhythmias in the atrium. To test our hypothesis, we modulated SERCA2a expression/function by SERCA2a overexpression using an adenoviral vector (Ad.SERCA2a) or with SERCA2a inhibition using Thapsigargin in isolated rat atrial myocytes.

## Methods

Experiments were carried out in accordance with the United States Public Health Service guidelines for the care and use of laboratory animals. IACUC approval at Case Western Reserve University "Gene Therapy for cardiac alternans" protocol number 2013–0131. Adult rat isolated atrial myocytes where divided into three treatment groups 1) Control, 2) SERCA2a overexpression (Ad.SERCA2a) and 3) SERCA2a inhibition (Thapsigargin, 1μm).

### Gene Delivery

Adenoviral vectors consisting of the CMV immediate/early promoter/enhancer, rabbit SERCA2a (Ad.SERCA2a) or no gene of interest then a second cassette for GFP reporter (Ad.GFP) were utilized as previously described.[8] *In-vivo* gene transfer was performed in a subset of male Sprague-Dawley rats using modified cross-clamping method as previously described.[8,9] *In-vitro* gene transfer was performed by incubating isolated rat myocytes with Ad.GFP (vector control) or Ad.SERCA2a.GFP for 24 hours.

### Isolation of rat atrial myocytes

Myocytes were isolated from rat hearts using modified enzymatic dispersion technique described previously.[10] The main modification was that to digest the hearts the enzyme solution containing collagenase II (0.8mg/ml) was perfused for 20 min. Myocytes were then incubated with Ad.SERCA2a (5 x 10^11^ p.f.u) or Ad.GFP (5 x 10^11^ p.f.u) for 24h. GFP-positive cells examined by fluorescence microscopy were used for experimental protocols.

### Ca^2+^ transient recordings

Intracellular Ca^2+^ transients were measured using the fluorescent Ca^2+^ indicator indo-1_AM_ as described previously.[11] Briefly, rat atrial myocytes were loaded with indo-1_AM_ by incubating them in Tyrode's containing indo-1_AM_ (2uM) (Molecular Probes) and 0.025% (wt/wt) Pluronic F-127 (Molecular Probes) for 20 min at room temperature. Myocytes were then warmed to 35°C and the indicator was excited at 350 nm and the emitted signals were measured simultaneously at 405 nm and 485 nm. The emission field was restricted to a single cell with the aid of an adjustable window. The background fluorescence recorded from a cell without indicator loaded at both wavelengths was subtracted from the signal recorded from the cell before the fluorescence ratio was calculated. The 405nm/485nm fluorescence ratio was used to monitor changes in [Ca^2+^]_i_. Myocytes were bathed in a chamber with Tyrode’s solution composed of (mmol/L) NaCl 137, KCl 5.4, CaCl_2_ 2, MgSO_4_ 1, Glucose 10, HEPES 10, pH to 7.35 with NaOH and were stimulated by field stimulation at a baseline pacing rate of 120 beat per minute (bpm). Following a period of stimulation to establish steady state, measurements were made for the subsequent 20 beats. This protocol was repeated at progressively faster rates to induce Ca^2+^ transient alternans.

Ca^2+^ transient parameters were defined referring to the methods described previously.[12] Diastolic Ca ^2+^ was defined as cytosolic Ca^2+^ level just prior to the onset of the Ca^2+^ transient. Amplitude of intracellular Ca^2+^ transient was calculated from the difference between peak and diastolic Ca^2+^. Duration of the intracellular Ca^2+^ transient was measured as the onset of the Ca^2+^ transient to the point of time when the transient decayed by 50%. To quantify the rate of recovery of intracellular Ca^2+^ to diastolic levels, the decay portion of the Ca^2+^ transient (from 30% to 100% of decline phase) was measured by the time constant (i.e. Tau) of a single exponential fit. SR content was estimated by the Ca^2+^ transient amplitude after caffeine pulse induced SR Ca release. Ca ^2+^ transient alternans threshold was defined as the pacing rate inducing Ca^2+^ transient amplitude alternans. Data acquisition were operated with an Axopatch 200B patch clamp amplifier controlled by a personal computer using a Digidata 1200 acquisition board driven by pCLAMP 7.0 software (Axon Instruments, Foster City, CA).

### Protein expression

Isolated atrial myocytes were divided into 2 dishes for Ad.SERCA2a or Ad.GFP infection. Paired samples were collected 24 hours post-gene transfer. Cells were spun down at 1.5rpm for 3 min and rinsed 3x in cold PBS. Cells were then lysed in RIPA lysis buffer plus complete protease inhibitor cocktail (Roche) and homogenized using a 25-gauge needle on ice. Lysates were run on 4–15% TGX gels (Bio-Rad) and blotted for SERCA2a (1:1000, Dr. Periasamy, Ohio State University), RyR (1:800, Affinity Bioreagents), Phospholamban (1:1000, Santa Cruz), NCX (1:1000, Swant) and actin (1:1000, Sigma-Aldrich). Comparisons were made within each animal (Rat 1 Ad.GFP myocytes vs. Ad.SERCA2a myocyte level of SERCA2a expression etc.).

### *In-vivo* arrhythmia susceptibility

3–5 days post-gene transfer, animals were anesthetized (Ketamine, Xylazine). Once fully anesthetized, a 5F quad catheter was advanced down the esophagus and 3 limb leads were attached (right arm, left arm, left leg). ECG recordings were recorded at baseline and pacing was verified before burst pacing to assess atrial arrhythmia susceptibility. Pacing protocols included 10–15 seconds of burst pacing followed by halted pacing to assess the rhythm. Burst pacing was performed at 160ms to 50ms decrementing by 10ms intervals to assess atrial arrhythmia susceptibility.

### Data Analysis & Statistics

Ca-ALT was measured by calculating the difference in amplitude on 2 consecutive beats, normalized to one of the beats and was defined to be present when Ca-ALT exceeded 10% of Ca^2+^ transient amplitude, as described previously.[12] Statistical differences were calculated using student’s t test and Chi-squared tests when appropriate.

## Results

### Increased SERCA2a expression does not suppress cardiac alternans in the atria

**Fig 1:**
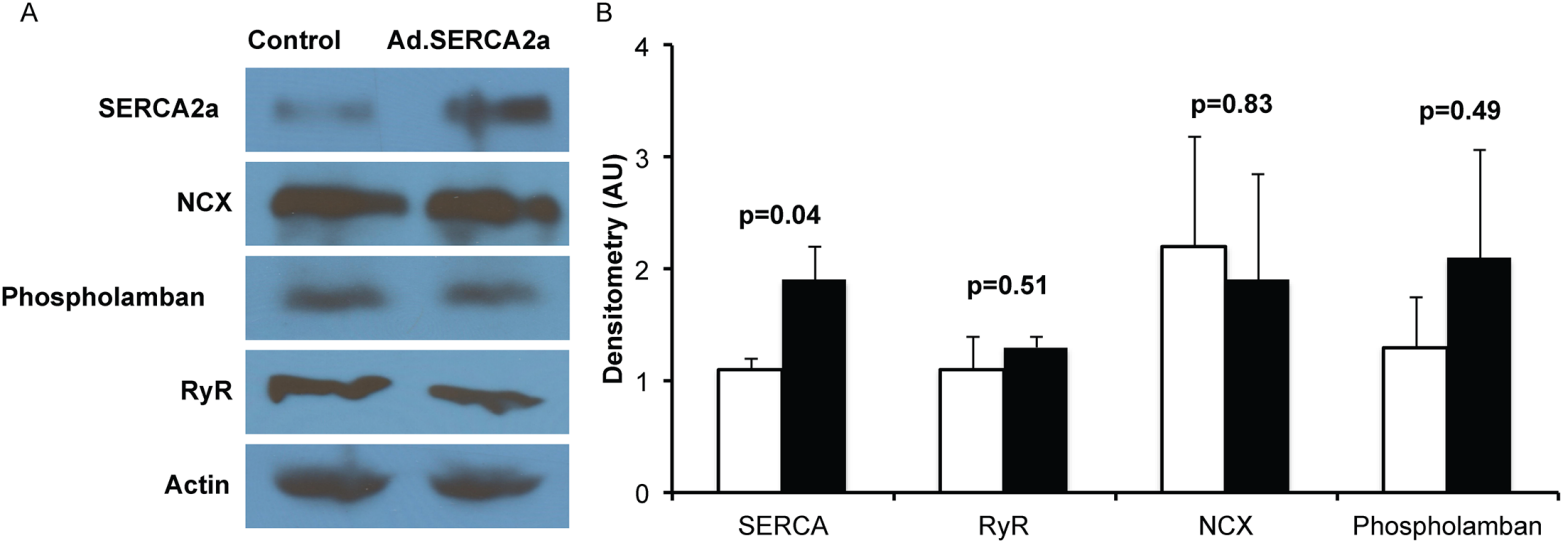
SERCA2a overexpression in isolated atrial myocytes. SERCA2a was overexpressed in isolated rat atrial myocytes for 24 hours. Ad.SERCA2a in isolated myocytes lead to an increase in SERCA2a expression without changing expression of other calcium cycling proteins Ryanodine Receptor (RyR), Na^+^/Ca^2+^ exchanger (NCX), and phospholamban. Westerns are depicted in A with summary data of SERCA2a overexpression (n = 4) and control myocytes (n = 4) in B.

**Fig 2:**
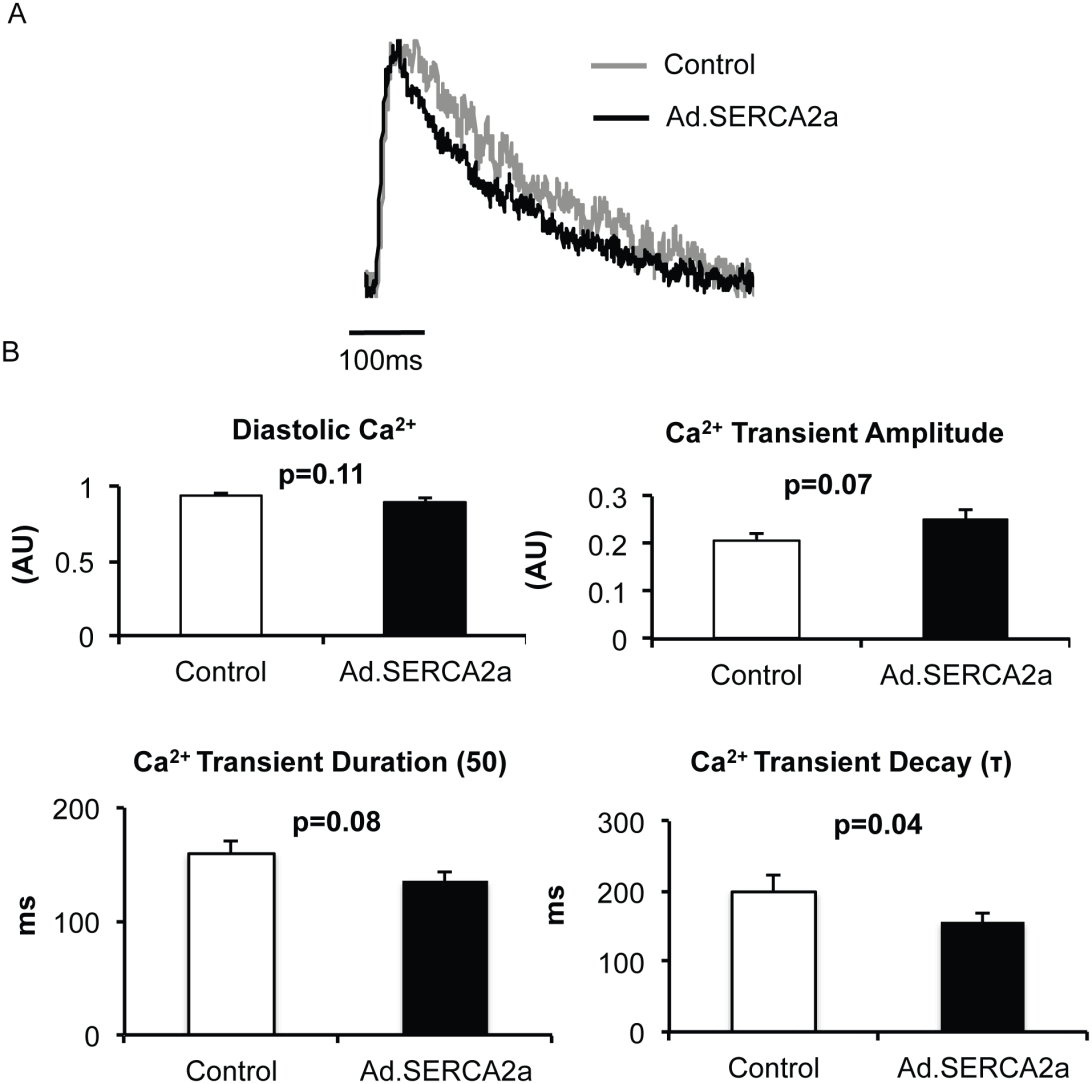
Ca^2+^ handling with Ad.SERCA2a expression. Intracellular Ca^2+^ transients were recorded in isolated rat atrial myocytes at a pacing rate of 120 bpm at 35°C. Representative calcium tracings are shown in A for a control myocyte (grey) and Ad.SERCA2a myocytes (black). Tracings are overlapped to depict differences. B Summary data from SERCA2a overexpression (n = 14) and control (n = 11) myocytes are shown. SERCA2a overexpression resulted in enhanced calcium reuptake (smaller Ca decay time constant (τ)). Ca^2+^ transient amplitude and duration showed nonsignificant trends of increased Ca^2+^ release and shorter duration.

**Fig 3:**
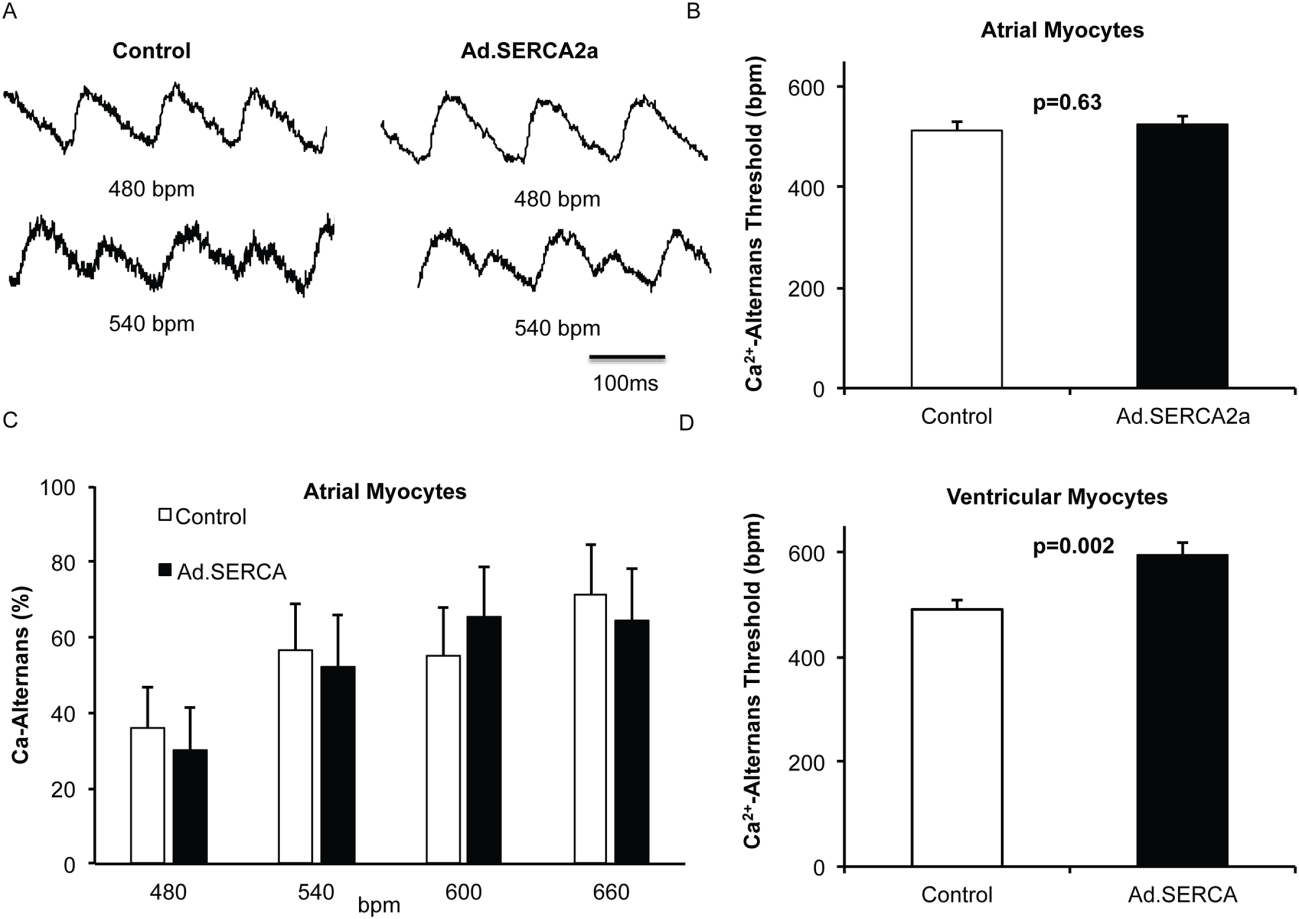
SERCA2a overexpression does not affect atrial Ca^2+^ alternans. Representative Ca^2+^ tracings of control (n = 11) and SERCA2a overexpression (Ad.SERCA2a, n = 12) atrial myocytes is depicted in A. Surprisingly, there was no difference in Ca^2+^ alternans threshold between control and Ad.SERCA2a expressing myocytes (B/C). Both began Ca-ALT as stimulation rate was increased from 480 bpm to 540 bpm. In contrast to atrial myocytes, ventricular myocytes overexpressing SERCA2a (n = 12) significantly increased Ca^2+^ alternans threshold compared to controls (n = 11, p = 0.002).

Adenoviral SERCA2a gene transfer in isolated rat atrial myocytes increased SERCA2a protein expression when compared to control myocytes (p<0.05, Fig 1). Importantly, we did not observe changes in other calcium handling proteins such as Na^+^-Ca^2+^ exchanger (NCX), ryanodine receptor (RyR) or phospholamban. As predicted, SERCA2a significantly reduced Ca^2+^ transient decay time (τ, p = 0.04, Fig 2). SERCA2a overexpression resulted in a nonsignificant trend toward decreased diastolic Ca^2+^ (p = 0.11) and increased Ca^2+^ transient amplitude (p = 0.07). Surprisingly, we did not observe a difference in Ca-ALT thresholds between control myocytes and Ad.SERCA2a expressing atrial myocytes (p = 0.63, Fig 3B/3C). In contrast, Ad.SERCA2a expressing ventricular myocytes did significantly increase Ca-ALT thresholds compared to control myocytes (p = 0.002, Fig 3D). This suggested that SERCA2a might not play a role in atrial alternans in the absence of disease.

### SERCA2a inhibition does not enhance cardiac alternans in the atria

**Fig 4:**
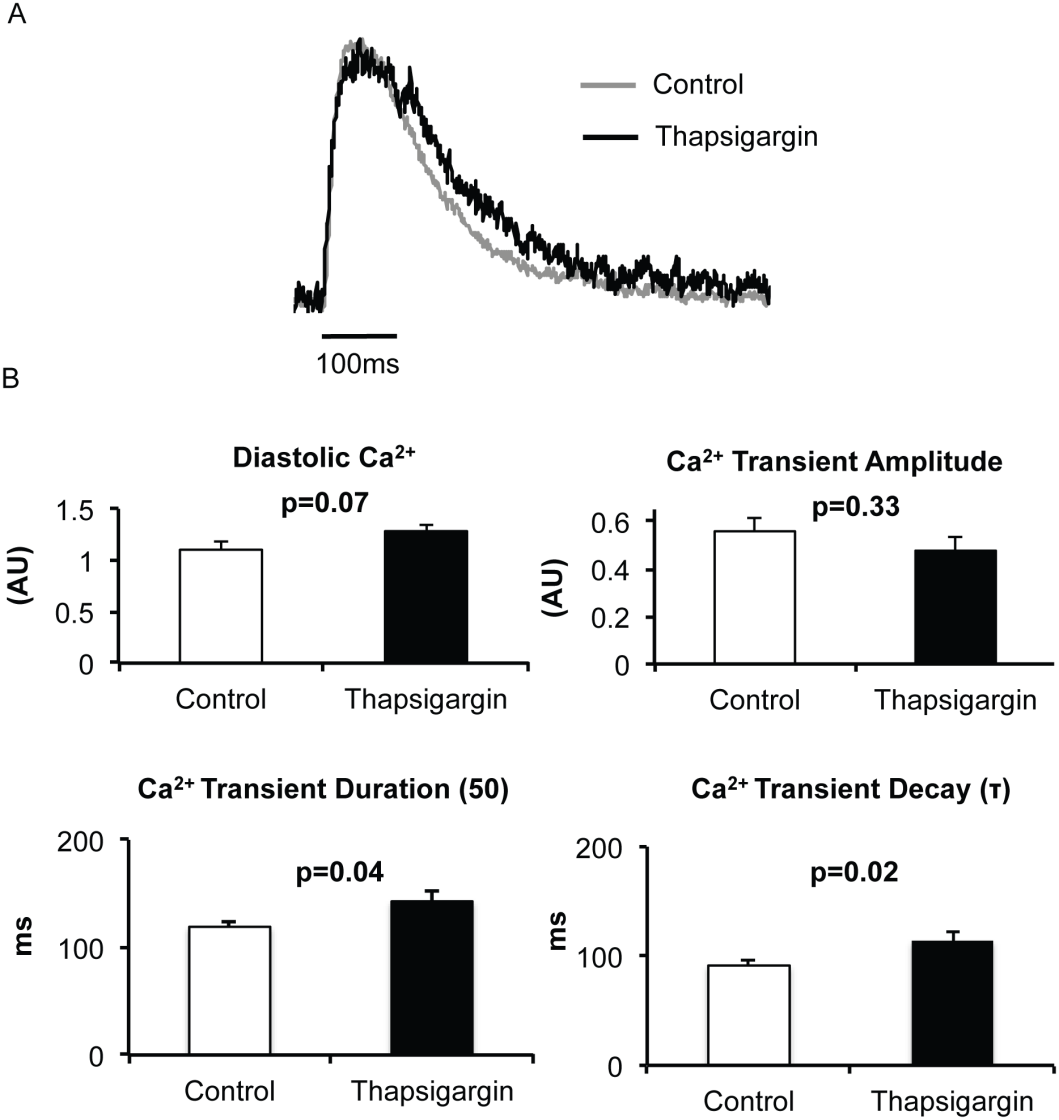
Ca^2+^ handling with SERCA2a inhibition. Intracellular Ca^2+^ transients were recorded in isolated rat atrial myocytes at a pacing rate of 120 bpm at 35°C. Representative calcium tracings are shown in A for a control myocyte (grey) and Thapsigargin, a SERCA2a inhibitor, treated myocytes (black). Tracings are overlapped to depict differences. B Summary data from control (n = 8) and Thapsigargin treated (n = 8) myocytes are shown. SERCA inhibition resulted in delayed calcium reuptake (larger Ca decay time constant (τ)) and prolonged Ca^2+^ transient durations. Diastolic Ca^2+^ had a nonsignificant trend toward increased and Ca^2+^ transient amplitude was not significant.

**Fig 5:**
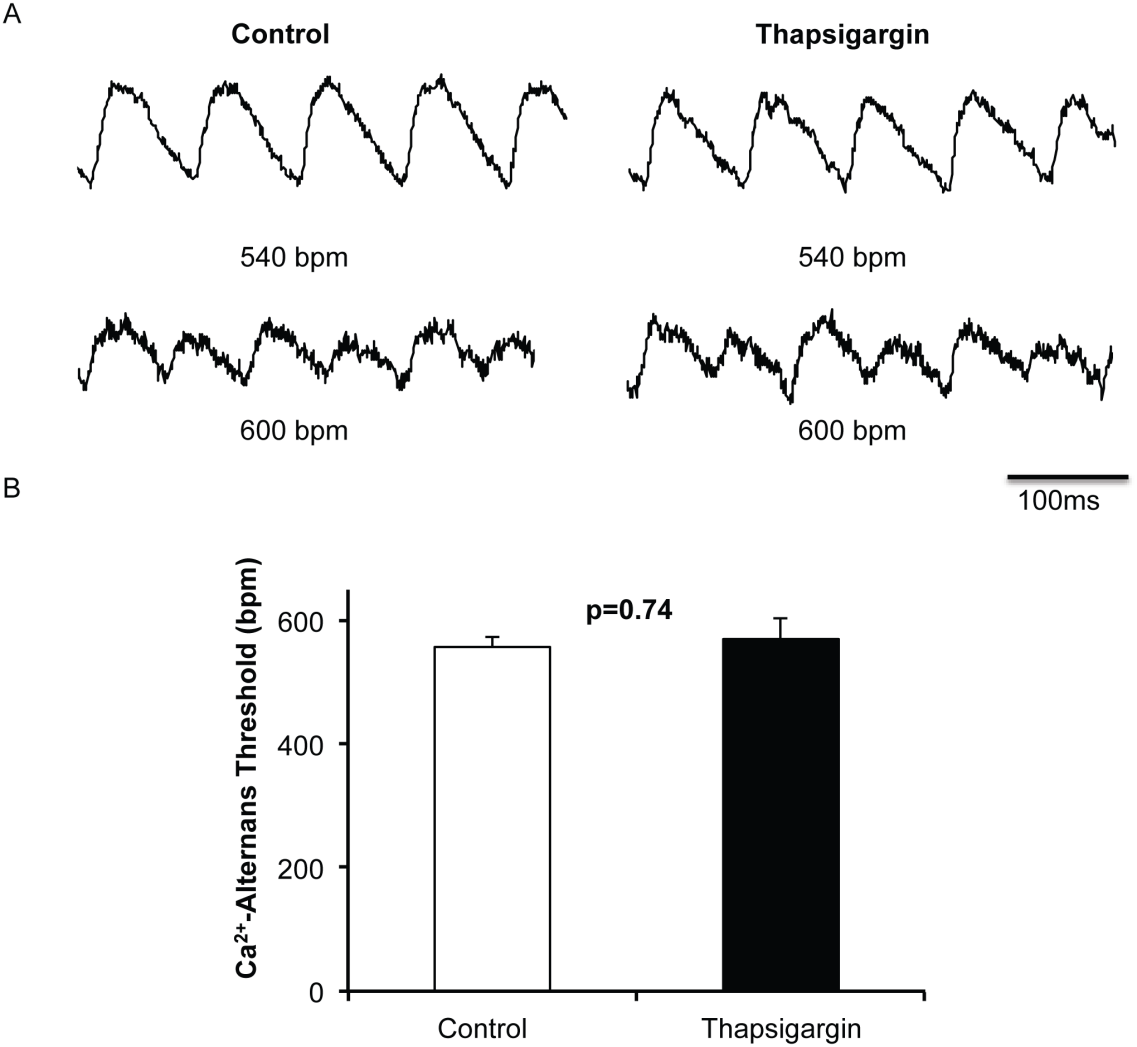
SERCA2a inhibition does not affect atrial Ca^2+^ alternans. Representative Ca^2+^ tracings of control (n = 8) and SERCA2a inhibited (Thapsigargin, n = 8) atrial myocytes is depicted in A. Surprisingly, there was no difference in Ca^2+^ alternans threshold between control and SERCA2a inhibited myocytes. Both began Ca-ALT as stimulation rate was increased to 600 bpm.

Based on the observation that SERCA2a overexpression does not contribute to atrial cardiac alternans we investigated the role of SERCA2a inhibition on cardiac alternans in the atria. Inhibition of SERCA2a with thapsigargin significantly increased calcium transient duration (p = 0.04), decreased the rate of calcium reuptake (τ, p = 0.02) and there was a nonsignificant trend toward increased diastolic calcium (p = 0.07, Fig 4). Consistent with our finding using SERCA2a overexpression, SERCA2a inhibition also did not alter Ca-ALT threshold when compared to control (p = 0.74, Fig 5). Taken together these results suggest that SERCA2a plays little role in atrial alternans in the absence of disease.

### SERCA2a increases arrhythmogenic substrates in isolated atrial myocytes

**Fig 6:**
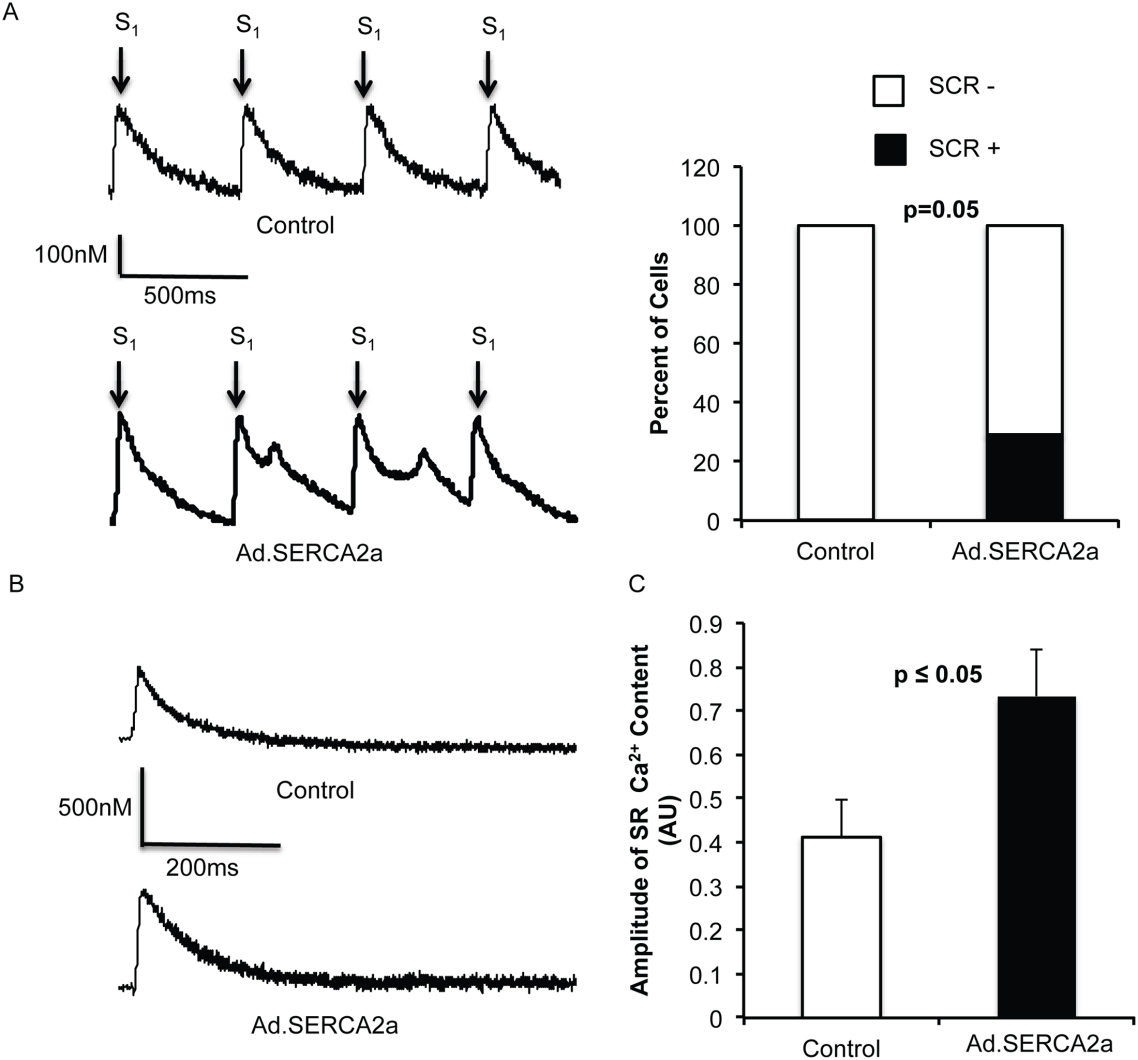
SERCA2a overexpression promotes spontaneous Ca^2+^ release. We observed a significant increase in premature SR Ca^2+^ release events (SCR) in Ad.SERCA2a expressing myocytes (control 0/11, Ad.SERCA2a 4/14, p = 0.05). Ca^2+^ traces are depicted in A, with S_1_ pacing shown with arrows. Control myocytes had no SCR (top panel), in comparison Ad.SERCA2a myocytes had SCR (bottom panel). Summary data is shown on the right. We stimulated with caffeine to determine SR content. We found Ad.SERCA2a resulted in increased SR content, which promoted SCR. Tracings are shown in B and summary data in C.

Interestingly, SERCA2a overexpression in atrial myocytes significantly increased premature SR Ca^2+^ release events (4 of 14 myocytes) when compared to control (0 of 11 myocytes, p = 0.04, Fig 6). Furthermore, Ad.SERCA2a expressing myocytes significantly increased SR calcium content (Fig 6B p<0.05), suggesting a likely mechanism for increased incidence of spontaneous Ca^2+^ release events in SERCA2a overexpressing atrial myocytes.

### SERCA2a overexpression may increase atrial arrhythmias *in-vivo*

**Fig 7:**
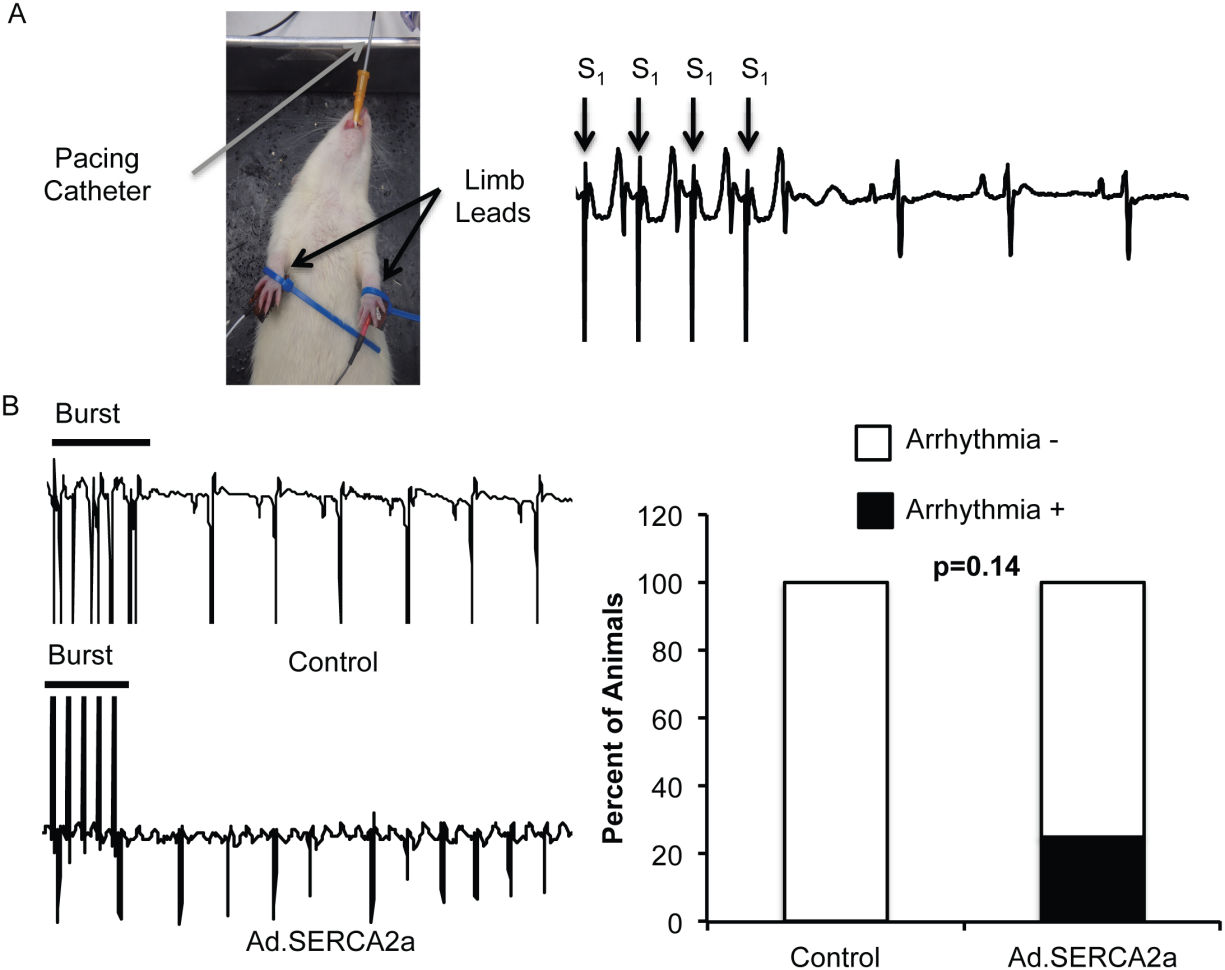
SERCA2a overexpression did not result in increased atrial arrhythmias. Since we observed significant increases in premature SR Ca^2+^ release events, we sought to determine if SERCA2a overexpression resulted in increased arrhythmias in vivo. In vivo gene transfer was accomplished through using aortic-pulmonary cross-clamp techniques while Ad.SERCA2a (n = 5) or Ad.GFP (n = 4 control) was injected. 3–5 days post gene-transfer, we transesophageally burst paced (catheter in grey) the atria as depicted in A. Standard limb leads were used to record ECGs. Pacing is depicted as S_1_. Burst pacing did not stimulate AF in control rats (B, top panel) but did stimulate AF in some Ad.SERCA2a rats (B, bottom panel). We observed 20% of animals to have AF with Ad.SERCA2a expression; however this was non-significant. Summary data is shown on the right in B.

Because we observed significant increases in arrhythmogenic substrates in isolated atrial myocytes, we wanted to test if Ad.SERCA2a gene transfer enhanced atrial arrhythmias *in-vivo*. We performed *in-vivo* gene transfer of Ad.SERCA2a in male Sprague-Dawley rats. Following 3–5 days post-gene transfer, a pacing catheter was advanced down the esophagus of anesthetized rats for transesophageal atrial pacing (Fig 7a). Atrial burst pacing induced atrial fibrillation (Fig 7b) in 20% of rats following Ad.SERCA2a gene transfer compared to no inducible atrial arrhythmias in Ad.GFP treated control animals (Fig 7c, p = 0.20).

## Discussion

Cardiac alternans in the atria has been postulated as an important mechanism of atrial fibrillation in humans.[6] Previously, we demonstrated that SERCA2a is an important molecular mechanism for the genesis of cardiac alternans in the ventricle. However, the role of SERCA2a in the development of cardiac alternans in the atria is less clear. A key finding of the present investigation is that in contrast to ventricular myocytes, SERCA2a does not modulate cardiac alternans in the atria. Furthermore, we found that SERCA2a overexpression can increase arrhythmogenic triggers in the atria, presumably from increased SR Ca^2+^ loading. These data suggest that in contrast to the ventricle, modulation of SERCA2a expression/activity in atrial myocytes does not alter susceptibility to cardiac alternans and may be arrhythmogenic secondary to increased premature SR Ca^2+^ release events.

### SERCA2a does not modulate cardiac alternans in the atria

Despite SERCA2a underlying the development of ventricular Ca-ALT, SERCA2a does not contribute to atrial alternans in healthy atria. In contrast to our finding, Tsai *et al*. stretched HL-1 cells, reduced SERCA2a expression and reported increased susceptibility to cardiac alternans, which was restored to baseline with SERCA2a gene transfer.[13] It is possible that differences in SR Ca^2+^ cycling kinetics exist between isolated adult atrial myocytes and HL-1 myocytes used by Tsai et al, accounting for the differences in the role of SERCA2a in modulating cardiac alternans. Further stretch induces hypertrophic signaling in addition to alterations in SERCA2a expression in atrial myocytes, which may contribute to changes in SR Ca^2+^ cycling kinetics between the two studies.[14] The strength of our study is that we utilized native atrial myocytes in contrast to immortal cell lines. Overall our data emphasize that SERCA2a does not contribute to atrial Ca-ALT under normal physiologic conditions.

In agreement with our findings atrial computer models predict increased SERCA2a uptake would not produce alterations in atrial calcium alternans.[15] Li *et al*. showed increased SERCA2a activity loaded the SR and maintained stable calcium waves without affecting Ca-ALT. However, this model also predicts SERCA2a inhibition would alter Ca-ALT through depletion of SR content limiting SR calcium release.[15] In the present study we did not observe alterations in Ca-ALT using Thapsigargin to inhibit SERCA2a activity. It is possible the degree of SR Ca^2+^ content depletion predicted by the model was greater than that produced by Thapsigargin in our study. Secondly, the model utilized by Li *et al*. was first developed based on ventricular calcium wave propagation and then altered to lack t-tubules. Calcium cycling kinetics and expression patterns of various calcium proteins differ between atrial and ventricular myocytes. Walden *et al*. found atrial myocytes have higher SR calcium content and enhanced calcium buffering capacity partly from greater SERCA2a activity.[16] Thus, overexpressing SERCA2a in atrial myocytes, which already have enhanced SERCA2a function, may not affect Ca-ALT because of already plateaued activity. [16] Further Walden *et al*. found atrial myocytes had smaller calcium transients than ventricular myocytes. Therefore although we inhibited SERCA2a, higher atrial SR content and lower calcium required for a Ca^2+^ transient may have lead to no changes in the development of Ca-ALT with SERCA2a inhibition.

Structural differences between ventricular and atrial myocytes may also explain why atrial SERCA2a overexpression would not alter atrial Ca-ALT thresholds. Small mammal and pathophysiologic atrial myocytes (i.e. heart failure, dilated atrium in large mammals) differ from ventricular myocytes in that they lack t-tubules.[17–19] This produces a fundamental change in the mechanism of electromechanical coupling. In contrast to ventricular myocytes, junctional SR-calcium propagates as a wave toward nonjunctional-SR, stimulating calcium release toward the central part of an atrial cell. Therefore, calcium waves are predicted to be strongly dependent upon junctional-SR content in the atrium. Overexpressing SERCA2a would serve to overload the SR, as shown with our data, but would not inhibit a calcium wave toward the central region of cell. This would allow for a cell to have minimal beat-to-beat variability in calcium cycling with increasing heart rates without the development of Ca-ALT.

The role of RyR2 in atrial alternans and arrhythmogenesis has been highlighted in several studies. Xie et al recently found that atrial ‘leaky’ RyR promoted Ca-ALT, Ca^2+^ waves and atrial arrhythmias.[20] Similarly, it was shown that RyR refractoriness lead to atrial Ca-ALT in a model of human atrial cells and rabbit atrial cells.[21,22] Others have also highlighted the importance of CaMKII hyperphosphorylation of RyR leading to Ca^2+^ dysregulation in AF.[23,24] Finally, it appears that in addition to Ca^2+^ dysregulation, structural remodeling or fibrosis significantly contributes to the development of alternans induced arrhythmias,[25] suggesting that sustained atrial arrhythmias require both structural and electrophysiologic remodeling. Despite the role RyR2 and atrial remodeling plays in atrial alternans, we importantly demonstrated ventricular SERCA2a-mediated alternans is absent in atrial cells.

### SERCA2a overexpression in the atrium increases arrhythmia triggers

In the present study we observed that atrial SERCA2a overexpression increased premature SR Ca^2+^ release events, most likely due to increased SR Ca^2+^ content. Increased SR calcium content greatly affects SR calcium release events and promotes spontaneous calcium release events.[26,27] Further, increased release of Ca^2+^ from the SR is an important trigger for atrial fibrillation, particularly in heart failure.[28] Therefore, we assessed if SERCA2a overexpression would increase atrial arrhythmias *in-vivo*. We observed a nonsignificant trend for increased inducible atrial arrhythmias in rats exposed to Ad.SERCA2a gene transfer compared to Ad.GFP control animals, suggesting atrial SERCA2a overexpression may increase AF arrhythmogenic triggers. We did not assess if SERCA2a overexpression increased atrial premature beats, which may have occurred as we caused increased SR content. Liang et al 2008 found isolated atrial myocytes from AF and normal patients had no difference in frequency of Ca^2+^ sparks,[29] suggesting frequency of Ca^2+^ sparks does not enhance atrial arrhythmias. Further we examined normal atria without any significant atrial remodeling. Sustained AF is dependent upon both electrical and structural remodeling such as atrial fibrosis, allowing for slowed conduction and reentry to develop.[30] In heart failure hearts, there is substantial atrial structural remodeling, suggesting SERCA2a overexpression in heart failure atria may be more proarrhythmic than we observed.

### Study Limitations

Limitations should be considered in extrapolating our data. Our studies were performed in normal atrial myocytes where SERCA2a function is not altered as in disease settings. However, we establish that SERCA2a does not modulate Ca-ALT in normal atrial myocytes suggesting SERCA2a function is not a key player in the development of Ca-ALT in the atrium as it is in the ventricular myocardium.

### Conclusions

Our data provide new insights into the potential mechanisms of atrial alternans and ultimately atrial fibrillation. In particular, we suggest that SERCA2a is not a key regulator of calcium alternans in the atrium, suggesting that RyR may be a more important determinant of atrial alternans. Importantly, SERCA2a overexpression in atrial myocytes may increase spontaneous SR Ca^2+^ release. In normal atrium, enhanced Ca^2+^ release events may not be proarrhythmic, but in remodeled atria such as in heart failure enhanced spontaneous SR Ca^2+^ release could prove to enhance the development of AF.

## Supporting Information

S1 Table SERCA2a western raw densitometry values underlying western for Fig 1. (DOCX)

S2 Table Raw patch-clamping values underlying Fig 2. (DOCX)

S3 Table Raw patch-clamping values underlying Fig 3. (DOCX)

S4 Table Raw patch-clamping values underlying Fig 4. (DOCX)

S5 Table Raw patch-clamping values underlying Fig 5. (DOCX)

S6 Table Raw numbers and patch-clamp values underlying Fig 6. (DOCX)

S7 Table Animal numbers underlying Fig 7. (DOCX)

## Abbreviations

AF: Atrial Fibrillation
SR: Sarcoplasmic Reticulum
Ca-ALT: Calcium Alternans
RyR: Ryanodine Receptor
Ad.SERCA2a: Adenoviral vector expressing SERCA2a
NCX: Na^+^/Ca^2+^ exchanger
