# Supplementary material for: Atrial SERCA2a Overexpression Has No Affect on Cardiac Alternans but Promotes Arrhythmogenic SR Ca2+ Triggers
Source: PLoS One. 2015 Sep 9;10(9):e0137359. doi: 10.1371/journal.pone.0137359 (PMC4564245; doi:10.1371/journal.pone.0137359)
Supplement: S1 Table — (DOCX) [file pone.0137359.s001.docx]

| S1 Table | | |
| --- | --- | --- |
| SERCA2a western densitometry | | |
|  | Control | AdSERCA2a |
|  | 0.942967929 | 2.467453543 |
|  | 0.884905748 | 1.180778477 |
|  | 1.061612316 | 2.38516881 |
|  | 1.385453536 | 1.603604526 |
