# Supplementary material for: Atrial SERCA2a Overexpression Has No Affect on Cardiac Alternans but Promotes Arrhythmogenic SR Ca2+ Triggers
Source: PLoS One. 2015 Sep 9;10(9):e0137359. doi: 10.1371/journal.pone.0137359 (PMC4564245; doi:10.1371/journal.pone.0137359)
Supplement: S2 Table — (DOCX) [file pone.0137359.s002.docx]

| S2 Table | | |
| --- | --- | --- |
| SERCA2a Diastolic Calcium | | |
|  | Control | AdSERCA2a |
|  | 0.863 | 0.725 |
|  | 0.975 | 0.807 |
|  | 0.978 | 0.824 |
|  | 0.93 | 0.886 |
|  | 0.959 | 0.919 |
|  | 0.953 | 0.924 |
|  | 1.023 | 1.075 |
|  | 0.887 | 0.992 |
|  | 0.919 | 0.897 |
|  | 0.921 | 0.863 |
|  | 0.854 | 0.836 |
|  |  | 0.863 |
|  |  | 0.994 |
|  |  | 0.922 |
| SERCA2a Calcium amplitude | | |
|  | Control | AdSERCA2a |
|  | 0.315 | 0.304 |
|  | 0.223 | 0.271 |
|  | 0.197 | 0.257 |
|  | 0.284 | 0.259 |
|  | 0.258 | 0.168 |
|  | 0.111 | 0.355 |
|  | 0.181 | 0.248 |
|  | 0.2 | 0.36 |
|  | 0.204 | 0.297 |
|  | 0.109 | 0.08 |
|  | 0.174 | 0.318 |
|  |  | 0.11 |
|  |  | 0.241 |
|  |  | 0.254 |
| SERCA2a Calcium Duration | | |
|  | Control | AdSERCA2a |
|  | 129 | 91 |
|  | 126 | 122 |
|  | 187 | 128 |
|  | 125 | 144 |
|  | 114 | 146 |
|  | 201 | 111 |
|  | 193 | 125 |
|  | 145 | 109 |
|  | 167 | 128 |
|  | 228 | 187 |
|  | 140 | 127 |
|  |  | 187 |
|  |  | 172 |
|  |  | 122 |
| SERCA2a tau | | |
|  | Control | AdSERCA2a |
|  | 120.199745 | 105.427711 |
|  | 141.748917 | 108.031265 |
|  | 220.834793 | 144.371567 |
|  | 135.733536 | 157.606583 |
|  | 127.479507 | 178.326294 |
|  | 369.792023 | 105.840294 |
|  | 219.859406 | 155.818909 |
|  | 177.038269 | 107.296127 |
|  | 210.812927 | 158.994308 |
|  | 303.645538 | 228.392014 |
|  | 167.327988 | 180.701492 |
|  |  | 235.000153 |
|  |  | 200.292267 |
|  |  | 128.671753 |
