# Supplementary material for: Atrial SERCA2a Overexpression Has No Affect on Cardiac Alternans but Promotes Arrhythmogenic SR Ca2+ Triggers
Source: PLoS One. 2015 Sep 9;10(9):e0137359. doi: 10.1371/journal.pone.0137359 (PMC4564245; doi:10.1371/journal.pone.0137359)
Supplement: S3 Table — (DOCX) [file pone.0137359.s003.docx]

| S3 Table | | |
| --- | --- | --- |
| SERCA2a alternans threshold | | |
|  | control | AdSERCA2a |
|  | 480 | 540 |
|  | 480 | 480 |
|  | 480 | 540 |
|  | 480 | 480 |
|  | 540 | 480 |
|  | 480 | 480 |
|  | 480 | 480 |
|  | 480 | 540 |
|  | 600 | 480 |
|  | 480 | 600 |
|  | 660 | 540 |
|  |  | 660 |
| SERCA2a ventricular alternans threshold | | |
|  | Control | AdSERCA2a |
|  | 540 | 420 |
|  | 360 | 660 |
|  | 540 | 540 |
|  | 480 | 600 |
|  | 480 | 660 |
|  | 420 | 600 |
|  | 480 | 540 |
|  | 480 | 540 |
|  | 600 | 720 |
|  | 540 | 600 |
|  | 480 | 600 |
|  |  | 660 |
