# Supplementary material for: Atrial SERCA2a Overexpression Has No Affect on Cardiac Alternans but Promotes Arrhythmogenic SR Ca2+ Triggers
Source: PLoS One. 2015 Sep 9;10(9):e0137359. doi: 10.1371/journal.pone.0137359 (PMC4564245; doi:10.1371/journal.pone.0137359)
Supplement: S4 Table — (DOCX) [file pone.0137359.s004.docx]

| S4 Table | | |
| --- | --- | --- |
| Thapsigargin Diastolic Calcium | | |
|  | Control | Thapsigargin treated |
|  | 0.932 | 1.46 |
|  | 1.01 | 1.078 |
|  | 1.106 | 1.06 |
|  | 1.439 | 1.198 |
|  | 0.912 | 1.425 |
|  | 1.39 | 1.329 |
|  | 0.936 | 1.32 |
|  | 1.09 | 1.391 |
| Thapsigargin Calcium amplitude | | |
|  | Control | Thapsigargin treated |
|  | 0.338 | 0.75 |
|  | 0.537 | 0.469 |
|  | 0.377 | 0.467 |
|  | 0.805 | 0.278 |
|  | 0.647 | 0.263 |
|  | 0.701 | 0.63 |
|  | 0.561 | 0.556 |
|  | 0.5 | 0.402 |
| Thapsigargin Calcium Duration | | |
|  | Control | Thapsigargin treated |
|  | 108 | 129 |
|  | 122 | 110 |
|  | 107 | 124 |
|  | 105 | 144 |
|  | 144 | 170 |
|  | 120 | 166 |
|  | 130 | 120 |
|  | 118 | 180 |
| Thapsigargin tau | | |
|  | Control | Thapsigargin treated |
|  | 90.578094 | 118.509644 |
|  | 86.388992 | 98.431412 |
|  | 79.936104 | 98.647179 |
|  | 91.523399 | 113.819092 |
|  | 97.41745 | 120.643517 |
|  | 72.633766 | 91.860321 |
|  | 119.757042 | 111.494225 |
|  | 92.327148 | 159.4112 |
