# Supplementary material for: Atrial SERCA2a Overexpression Has No Affect on Cardiac Alternans but Promotes Arrhythmogenic SR Ca2+ Triggers
Source: PLoS One. 2015 Sep 9;10(9):e0137359. doi: 10.1371/journal.pone.0137359 (PMC4564245; doi:10.1371/journal.pone.0137359)
Supplement: S5 Table — (DOCX) [file pone.0137359.s005.docx]

| S5 Table | | |
| --- | --- | --- |
| Thapsigargin alternans threshold | | |
|  | Control | Thapsigargin |
|  | 540 | 480 |
|  | 540 | 660 |
|  | 600 | 540 |
|  | 480 | 480 |
|  | 600 | 480 |
|  | 540 | 720 |
|  | 600 | 600 |
|  |  | 600 |
