# Supplementary material for: Atrial SERCA2a Overexpression Has No Affect on Cardiac Alternans but Promotes Arrhythmogenic SR Ca2+ Triggers
Source: PLoS One. 2015 Sep 9;10(9):e0137359. doi: 10.1371/journal.pone.0137359 (PMC4564245; doi:10.1371/journal.pone.0137359)
Supplement: S6 Table — (DOCX) [file pone.0137359.s006.docx]

| S6 Table | | |
| --- | --- | --- |
| SERCA2a spontaneous calcium release events + cells | | |
|  | Control | AdSERCA2a |
|  | 0 of 11 | 5 of 14 |
| SERCA SR calcium content | | |
|  | Control | AdSERCA2a |
|  | 0.181 | 0.7 |
|  | 0.1 | 0.383 |
|  | 0.771 | 0.34 |
|  | 0.63 | 0.85 |
|  | 0.6 | 0.521 |
|  | 0.359 | 1.018 |
|  | 0.467 | 0.687 |
|  | 0.187 | 1.478 |
|  |  | 0.469 |
|  |  | 0.88 |
