# Supplementary material for: Atrial SERCA2a Overexpression Has No Affect on Cardiac Alternans but Promotes Arrhythmogenic SR Ca2+ Triggers
Source: PLoS One. 2015 Sep 9;10(9):e0137359. doi: 10.1371/journal.pone.0137359 (PMC4564245; doi:10.1371/journal.pone.0137359)
Supplement: S7 Table — (DOCX) [file pone.0137359.s007.docx]

| S7 Table | | |
| --- | --- | --- |
| SERCA2a atrial arrhythmia animals | | |
|  | control | AdSERCA2a |
|  | 0 of 4 | 1 of 5 |
